# Intracerebroventricular Treatment with 2-Hydroxypropyl-β-Cyclodextrin Decreased Cerebellar and Hepatic Glycoprotein Nonmetastatic Melanoma Protein B (GPNMB) Expression in Niemann–Pick Disease Type C Model Mice

**DOI:** 10.3390/ijms22010452

**Published:** 2021-01-05

**Authors:** Madoka Fukaura, Yoichi Ishitsuka, Seiichi Shirakawa, Naoki Ushihama, Yusei Yamada, Yuki Kondo, Toru Takeo, Naomi Nakagata, Keiichi Motoyama, Taishi Higashi, Hidetoshi Arima, Yuki Kurauchi, Takahiro Seki, Hiroshi Katsuki, Katsumi Higaki, Muneaki Matsuo, Tetsumi Irie

**Affiliations:** 1 Department of Clinical Chemistry and Informatics, Graduate School of Pharmaceutical Sciences, Kumamoto University, 5-1 Oe-honmachi, Chuo-ku, Kumamoto 862-0973, Japan; 171y2002@st.kumamoto-u.ac.jp (M.F.); shishimo13@gmail.com (S.S.); mrbruamdp.dada70ki@docomo.ne.jp (N.U.); yusei_yamada@med.miyazaki-u.ac.jp (Y.Y.); ykondo@kumamoto-u.ac.jp (Y.K.); 2 Program for Leading Graduate Schools “HIGO (Health Life Science: Interdisciplinary and Glocal Oriented) Program,” Kumamoto University, 5-1 Oe-honmachi, Chuo-ku, Kumamoto 862-0973, Japan; 3 Division of Reproductive Engineering, Center for Animal Resources and Development (CARD), Kumamoto University, 2-2-1 Honjo, Kumamoto 860-0811, Japan; takeo@kumamoto-u.ac.jp (T.T.); nakagata@gpo.kumamoto-u.ac.jp (N.N.); 4 Department of Physical Pharmaceutics, Graduate School of Pharmaceutical Sciences, Kumamoto University, 5-1 Oe-honmachi, Chuo-ku, Kumamoto 862-0973, Japan; motoyama@kumamoto-u.ac.jp (K.M.); higashit@kumamoto-u.ac.jp (T.H.); 5 Laboratory of Evidence-based Pharmacotherapy, Daiichi University of Pharmacy, 22-1 Tamagawa-machi, Minami-ku, Fukuoka 815-8511, Japan; h-arima@daiichi-cps.ac.jp; 6 Department of Chemico-Pharmacological Sciences, Graduate School of Pharmaceutical Sciences, Kumamoto University, 5-1 Oe-honmachi, Chuo-ku, Kumamoto 862-0973, Japan; kurauchy@kumamoto-u.ac.jp (Y.K.); takaseki@kumamoto-u.ac.jp (T.S.); hkatsuki@gpo.kumamoto-u.ac.jp (H.K.); 7 Research Initiative Center, Organization for Research Initiative and Promotion, Tottori University, 86 Nishi-cho, Yonago 683-8503, Japan; kh4060@tottori-u.ac.jp; 8 Department of Pediatrics, Faculty of Medicine, Saga University, 5-1-1 Nabeshima, Saga 849-8501, Japan; matsuo@cc.saga-u.ac.jp

**Keywords:** Niemann–Pick disease type C, 2-hydroxypropyl-β-cyclodextrin, glycoprotein nonmetastatic melanoma B

## Abstract

Niemann–Pick disease type C (NPC) is a recessive hereditary disease caused by mutation of the *NPC1* or *NPC2* gene. It is characterized by abnormality of cellular cholesterol trafficking with severe neuronal and hepatic injury. In this study, we investigated the potential of glycoprotein nonmetastatic melanoma protein B (GPNMB) to act as a biomarker reflecting the therapeutic effect of 2-hydroxypropyl-β-cyclodextrin (HP-β-CD) in an NPC mouse model. We measured serum, brain, and liver expression levels of GPNMB, and evaluated their therapeutic effects on NPC manifestations in the brain and liver after the intracerebroventricular administration of HP-β-CD in *Npc1* gene-deficient (*Npc1*^−/−^) mice. Intracerebroventricular HP-β-CD inhibited cerebellar Purkinje cell damage in *Npc1*^−/−^ mice and significantly reduced serum and cerebellar GPNMB levels. Interestingly, we also observed that the intracerebral administration significantly reduced hepatic GPNMB expression and elevated serum ALT in *Npc1*^−/−^ mice. Repeated doses of intracerebroventricular HP-β-CD (30 mg/kg, started at 4 weeks of age and repeated every 2 weeks) drastically extended the lifespan of *Npc1*^−/−^ mice compared with saline treatment. In summary, our results suggest that GPNMB level in serum is a potential biomarker for evaluating the attenuation of NPC pathophysiology by intracerebroventricular HP-β-CD treatment.

## 1. Introduction

Niemann–Pick disease type C (NPC) is an autosomal recessive disorder that develops as a result of *NPC1* or *NPC2* gene mutation (95% and 5% of patients, respectively) and has been identified as a severe disease associated with lysosomal dysfunction [1]. The transmembrane protein NPC1 and the soluble luminal protein NPC2 play important roles in transporting cellular cholesterol from lysosomes/late endosomes to other organelles, such as the endoplasmic reticulum. NPC1/2 protein dysfunction leads to the accumulation of free cholesterol and depletion of esterified cholesterol in cells throughout the body of model animals and patients [2,3]. Animals and patients with NPC experience systemic symptoms, including neurological dysfunction and severe liver dysfunction [4]. The symptoms of NPC, particularly neurological dysfunction, progressively worsen in most patients, so there is an urgent need to develop an effective cure.

2-Hydroxypropyl-β-cyclodextrin (HP-β-CD), a cyclic oligosaccharide inclusion complex with lipophilic compounds, including cholesterol, is widely used as a food additive and solubilizing agent in pharmaceuticals. Some studies have indicated that HP-β-CD has potential benefits against manifestations of NPC in cellular and animal models [5,6,7]. Recently, HP-β-CD was compassionately administered to NPC patients [8,9] and is in ongoing clinical trials in the United States and the European Union [10]. However, the lack of biomarkers that accurately reflect the pathogenesis of NPC still makes it difficult to determine the effectiveness of treatment.

Glycoprotein nonmetastatic melanoma protein B (GPNMB) was originally identified as a membrane protein, but it has been demonstrated that soluble GPNMB is released from astrocytes, melanocytes, and breast cancer cells [11]. Recently, GPNMB mRNA expression was reported to be increased in the brain, liver, and spleen of *Npc1*^−/−^ mice [12]. Marques et al. observed a significant elevation of blood GPNMB levels in NPC mice and patients [13]. They also found that miglustat (N-butyl-1-deoxynojirimycin), a glucosylceramide synthase and an approved drug for NPC patients in Europe and Japan, reduced *Gpnmb* mRNA expression induced by U18666A in the RAW 264.7 murine macrophage cell line, probably through the inhibition of glucosylceramide synthase but not cholesterol synthesis. Given this background, GPNMB has been anticipated to be a candidate biomarker for characterizing and evaluating the disease condition in NPC [13]. In this study, we investigated the potential of GPNMB to act as a serum biomarker during HP-β-CD treatment and examined the relationship between GPNMB expression and NPC symptoms in model mice treated with HP-β-CD.

## 2. Results

### 2.1. Protective Effects of Intracerebroventricular HP-β-CD Treatment on Neuronal Injury in Npc1-Deficient Mice

**Figure 1:**
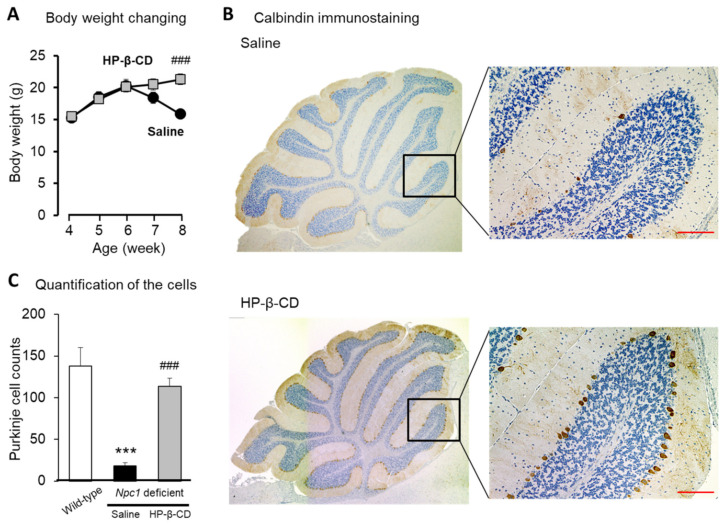
Potential of the intracerebroventricular administration of 2-hydroxypropyl-β-cyclodextrin (HP-β-CD) to protect against reductions in body weight (**A**) and cerebellar Purkinje cells (B and C) in *Npc1*^−/−^ mice. HP-β-CD was administered at 21.4 μmol/kg in mice at 4 and 6 weeks of age. We recorded the changes in body weight once a week from 4 to 8 weeks old. We collected samples from the whole brain, liver, and blood at 8 weeks and 3 days of age and analyzed them. Histological images of the mouse cerebellum (**B**). Scale bar: 100 μm. Quantitative evaluation of calbindin-positive cells (**C**). Data are presented as the mean ± S.E. (*n* = 8–11). *** *p* < 0.001 compared with the wild-type group. ### *p* < 0.001 compared with the saline group.

To confirm the potential of HP-β-CD administered intracerebroventricularly to attenuate the neurological deficits in the cerebellum, calbindin immunostaining was performed in *Npc1*^−/−^ mice [14,15]. As shown in Figure 1A, the body weight of *Npc1*^−/−^ mice was significantly reduced in the saline-treated group from 7–8 weeks old, although before the start of this reduction, their growth appeared to be normal. Intracerebroventricular injections of HP-β-CD (21.4 μmol/kg, at 4 and 6 weeks of age, total two times for each mouse) suppressed the body weight reduction.

In the histological evaluation of Purkinje cell loss in the mouse cerebellum, calbindin immunostaining was performed. In the saline-treated *Npc1*^−/−^ mouse group, few calbindin-positive cells were found compared with the level in the saline-treated wild-type group in the observation conducted at 8 weeks of age. In contrast, a large number of calbindin-positive cells was detected in the HP-β-CD-treated *Npc1*^−/−^ group, which was significantly different from that in the saline-treated *Npc1*^−/−^ group (Figure 1C).

### 2.2. Evaluation of Soluble GPNMB (sGPNMB) Levels upon Intracerebroventricular HP-β-CD Treatment in Npc1^−/−^ Mice

**Figure 2:**
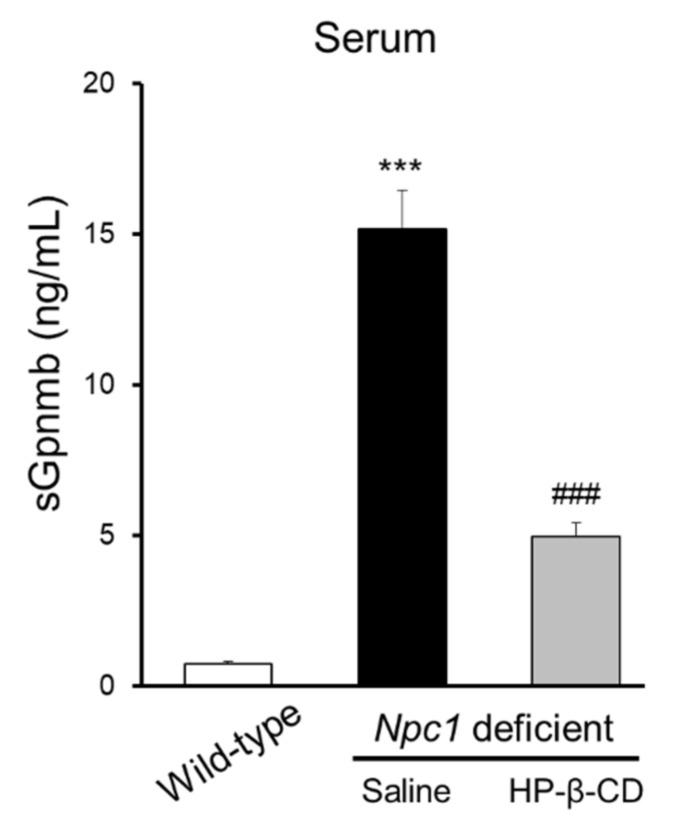
Suppression of serum soluble glycoprotein nonmetastatic melanoma protein B (sGPNMB) levels in *Npc1*^−/−^ mice treated with 2-hydroxypropyl-β-cyclodextrin (HP-β-CD) intracerebroventricularly. Levels of sGPNMB in mouse serum were assayed by ELISA. Data are presented as the mean ± S.E. (*n* = 8–11). *** *p* < 0.001 compared with the wild-type group. ### *p* < 0.001 compared with the saline group.

In a previous study, Marques et al. reported that soluble GPNMB was increased in *Npc1*^−/−^ mouse serum [13]. Therefore, we investigated how the intracerebroventricular administration of HP-β-CD affects sGPNMB in *Npc1*^−/−^ mouse serum. As shown in Figure 2, a significant increase in sGPNMB in serum was observed in saline-treated *Npc1*^−/−^ mice. Intracerebroventricular injection of HP-β-CD significantly reduced these abnormal increases.

### 2.3. Attenuating Effect of Intracerebroventricular Injection of HP-β-CD on Abnormal Increases of GPNMB in Npc1^−/−^ Mouse Cerebellum

**Figure 3:**
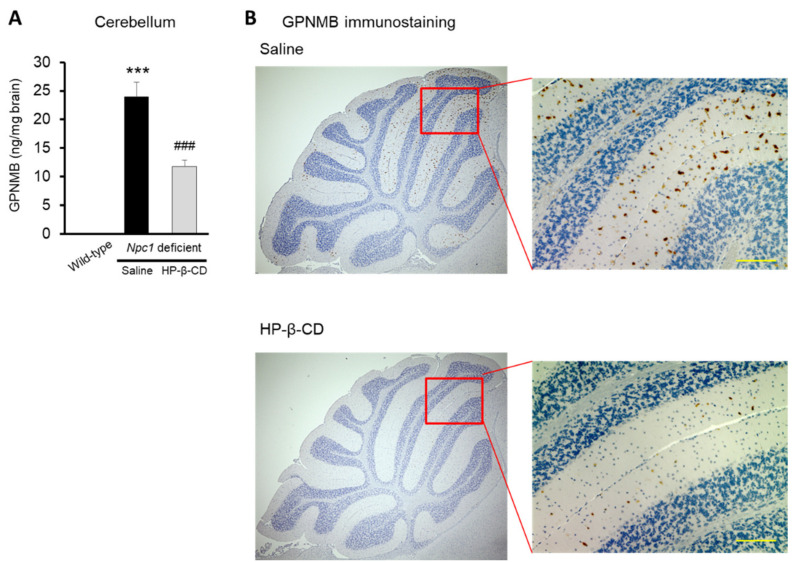
Intracerebroventricular treatment with 2-hydroxypropyl-β-cyclodextrin (HP-β-CD) attenuated the abnormal increase of glycoprotein nonmetastatic melanoma protein B (GPNMB) expression in *Npc1*^−/−^ mice. Assays of GPNMB levels in brain using ELISA (**A**) and immunohistochemical staining of GPNMB (**B**) were performed. Scale bar: 100 μm. Data are presented as the mean ± S.E. (*n* = 4–7). *** *p* < 0.01 compared with the wild-type group. ### *p* < 0.001 compared with the saline group.

Next, we examined the changes in GPNMB levels in the cerebellum after the intracerebroventricular administration of HP-β-CD in *Npc1* mice. As shown in Figure 3A, there was a pronounced increase in GPNMB levels in the cerebellum in saline-treated *Npc1*^−/−^ mice. In contrast, this increase was significantly inhibited in *Npc1*^−/−^ mice administered HP-β-CD intracerebroventricularly. Representative histological images (osteoactivin/GPNMB immunostaining) of *Npc1*^−/−^ mouse cerebellum are shown in Figure 3B. In saline-treated *Npc1*^−/−^ mice, GPNMB -positive cells were found in large areas of the cerebellum, whereas there were only a few positive cells in *Npc1*^−/−^ mice treated with HP-β-CD intracerebroventricularly.

### 2.4. Hepatoprotective Effects of Intracerebroventricular HP-β-CD Treatment in Npc1-Deficient Mice

**Figure 4:**
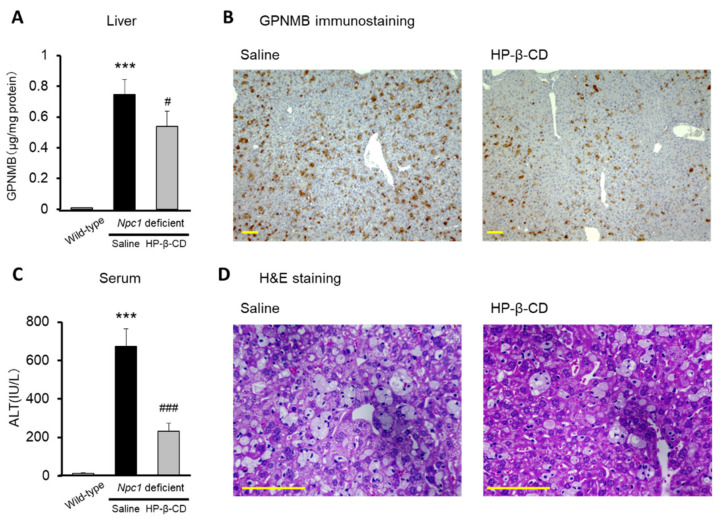
Attenuating effects of intracerebroventricular 2-hydroxypropyl-β-cyclodextrin (HP-β-CD) treatment on liver manifestations in *Npc1*^−/−^ mice. Glycoprotein nonmetastatic melanoma protein B (GPNMB) levels in liver homogenate (**A**), GPNMB immunohistochemistry in liver (**B**), serum alanine aminotransferase (ALT) levels (**C**), and hematoxylin-and-eosin (H&E)-stained hepatic histology (**D**) are shown. Scale bar: 100 μm for GPNMB and H&E staining. Each bar represents the mean ± S.E.M. (*n* = 8–11). *** *p* < 0.001 compared with the wild-type group. # *p* < 0.05, ### *p* < 0.001 compared with the saline group.

We evaluated the effect of intracerebroventricular treatment with HP-β-CD on hepatic changes in *Npc1*^−/−^ mice. The GPNMB level in the liver was elevated in saline-treated *Npc1*^−/−^ mice, but this increase was significantly inhibited in mice treated with HP-β-CD intracerebroventricularly (Figure 4A). Representative histological images (osteoactivin/GPNMB immunostaining) of *Npc1*^−/−^ mouse livers are shown in Figure 4B. GPNMB positive cells were quantified from the mouse liver samples (Supplemental Figure S1). An abnormal increase in serum transaminase levels was also observed in saline-treated *Npc1*^−/−^ mice (approximately 650 IU/L for alanine aminotransferase (ALT)), but this increase was significantly attenuated by HP-β-CD treatment (Figure 4C). In addition, representative histopathological images (hematoxylin and eosin (H&E) staining) of the livers of *Npc1*^−/−^ mice are shown in Figure 4D. Extensive vacuolated hepatocytes and Kupffer cells were observed in saline-treated *Npc1*^−/−^ mice. Notably, HP-β-CD administered intracerebroventricularly ameliorated at least some of the pathological changes in these livers. This result is consistent with the results obtained by Vite et al. using an NPC cat model [16].

### 2.5. Repeated Doses of Intracerebroventricular HP-β-CD Drastically Prolonged the Lifespan of Npc1-Deficient Mice

**Figure 5:**
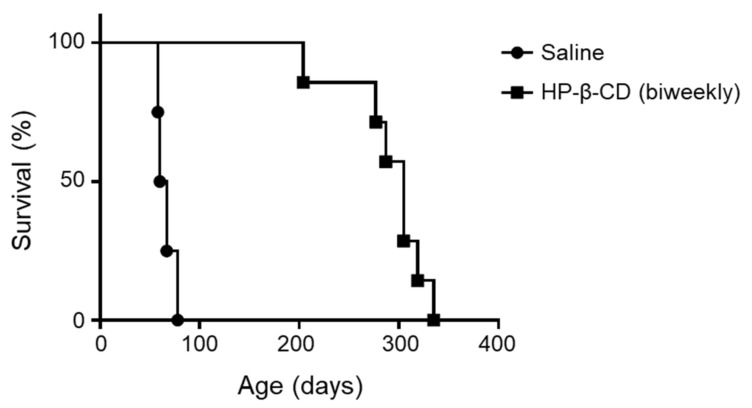
Life-prolonging effect of intracerebroventricular administration of 2-hydroxypropyl-β-cyclodextrin (HP-β-CD) in *Npc1*^−/−^ mice. HP-β-CD treatment (21.4 μmol/kg) was started at 4 weeks of age, with repeated injection into the mice every 2 weeks. Kaplan–Meier survival curves for the *Npc1*^−/−^ mice are shown (*n* = 4 for the saline-treated group (circle) and *n* = 7 for the HP-β-CD (biweekly) group (square)). A significant difference between the two groups was identified by the log-rank test (*p* < 0.01).

Previous studies have indicated that a single treatment of HP-β-CD administered intracerebroventricularly could ameliorate shortened lifespan, disrupt motor function, and prevent Purkinje cell loss, among other features, in *Npc1*^−/−^ mice [17]. However, the lasting duration of the preventive effects of intracerebroventricular HP-β-CD remains unclear. In this study, we evaluated the effect of repeated doses of intracerebroventricular HP-β-CD every 2 weeks on survival time in *Npc1*^−/−^ mice. We started the first dose of HP-β-CD (30 mg/kg) at 4 weeks of age and then administered it every 2 weeks intracerebroventricularly. The results of the Kaplan–Meier analysis are shown in Figure 5. The HP-β-CD group showed a significantly longer survival time than the saline-treated group, and the median survival times were 63.5 and 305 days, respectively.

## 3. Discussion

HP-β-CD treatment through the intracerebroventricular or intrathecal route has been reported as a possible attractive therapy for NPC neuronal injury [9,16]. There is also a need to develop a disease-related biomarker that can indicate the therapeutic effects of HP-β-CD. GPNMB has been suggested as a potential biomarker for the visceral pathological changes in NPC [13]. However, it remains unknown whether the treatment of HP-β-CD would change the GPNMB secretion and expression in NPC. In this study, we demonstrated that intracerebroventricular treatment of HP-β-CD significantly decreased GPNMB in serum and cerebellum, as well as prevented cerebellar Purkinje cell loss in *Npc1*^−/−^ mice. Interestingly, we also observed that intracerebroventricular treatment with HP-β-CD significantly reduced hepatic GPNMB expression, serum ALT elevation, and histological changes in *Npc1*^−/−^ mice. These results indicated that HP-β-CD treatment can reduce systemic GPNMB expression, including in the brain and liver, the main symptomatic organs in NPC, and can alleviate NPC symptoms.

Previously, Marques et al. [13] reported high levels of GPNMB in the blood of *Npc1*^−/−^ mice and NPC patients. They also demonstrated the reducing effect of miglustat, a glucosylceramide synthase inhibitor, on GPNMB protein and mRNA expression in a U18666A-induced NPC phenotype model using RAW264.7 murine macrophage cells. In this experiment, although there was a significant reduction in the production of glycosphingolipids, such as ceramide, glucosylceramide, and lactosylceramide, upon treatment with miglustat, no effect on cholesterol accumulation was found in U18666A-treated RAW264.7 cells. Based on these results, it was concluded that GPNMB is upregulated in NPC mice and patients, most likely due to glycosphingolipid accumulation. GPNMB is thus a potential candidate for assessing the efficacy of miglustat. In this study, we found significant elevation of serum GPNMB levels in *Npc1*^−/−^ mice compared with that in wild-type mice, which is consistent with the results reported by Marques et al. In addition, this study showed that the intracerebroventricular administration of HP-β-CD, a biocompatible cholesterol solubilizer, drastically reduced serum GPNMB levels in *Npc1*^−/−^ mice. We also demonstrated the neuroprotective effect of HP-β-CD treatment at the same time, and it seemed to be correlated with the serum GPNMB levels. These results suggest that serum GPNMB level is a potential biomarker for evaluating the effects of HP-β-CD in attenuating the manifestations of NPC.

In this study, we observed significant increases of the GPNMB concentration in cerebellar homogenate and the number of GPNMB-positive cells upon histological analysis of the cerebellum in *Npc1*^−/−^ mice. Treatment with HP-β-CD intracerebroventricularly reduced the cerebellar expression of GPNMB as well as its serum level. Previous studies reported that intracerebroventricular or intracisternal treatment with HP-β-CD drastically attenuated cerebellar injury in NPC animal models [16,17]. Neurological improvement was observed in NPC patients treated with HP-β-CD intracerebroventricularly or intrathecally [9]. In contrast, intravenous treatment with HP-β-CD for NPC showed little effect on the neurological deficits in patients with this condition, although partial attenuation of hepatosplenomegaly was observed [8]. These reports suggest that HP-β-CD applied intracerebroventricularly can achieve more potent neuronal protection than its application intravenously. However, intracerebroventricular HP-β-CD has been considered to have no effect on hepatic changes in NPC compared with systemic treatment. We observed a statistically significant reduction of hepatic GPNMB expression upon intracerebroventricular HP-β-CD treatment in *Npc1*^−/−^ mice. Furthermore, the intracerebroventricular HP-β-CD also reduced serum ALT levels in the mouse model. Although further study is warranted, these findings suggest that the reduction of GPNMB expression in serum reflects not only neuronal protection but also hepatic improvement of NPC manifestations during the intracerebroventricular administration of HP-β-CD.

In this study, intracerebroventricular HP-β-CD treatment showed attenuating effects on NPC-related neuronal and liver injury and effects of reducing cerebellar, hepatic, and serum levels of GPNMB in *Npc1*^−/−^ mice. However, this study had several limitations. First, the pathophysiological roles of GPNMB remain unclear. Previous studies have suggested significant increases of GPNMB in other lysosomal diseases, such as Gaucher disease [18] and lipid metabolic disorders [19], in animal models as well as in NPC [13]. In the case of hepatic fibrosis models, the introduction of a *Gpnmb* transgene and *Gpnmb* overexpression attenuated the hepatic injury and lipid accumulation, in contrast to the adverse effects on hepatic disorder of *Gpnmb* gene knockout, in diet-induced [20] and carbon-tetrachloride-induced mouse models [21]. Increasing levels of GPNMB may exert attenuating effects on NPC disease, although the precise roles are still unclear. Further study will be needed to clarify this point. A second limitation is that the localization of GPNMB expression in NPC cerebellum and liver remain unclear. Gabriel et al. [22] demonstrated a significant increase in *Gpnmb* gene transcription in adipose tissue macrophages in obese mice and suggested that GPNMB is a novel marker for obesity-induced adipose tissue macrophage infiltration. In addition, Martijn et al. [11] reported that GPNMB would be a biomarker of stressed macrophages in lysosomal storage diseases. In our morphological observation, GPNMB appeared to be strongly expressed in Kupffer cells and microglial cells in NPC mouse liver and cerebellum. However, to clearly demonstrate the localization of GPNMB in NPC pathology, further experiments using costaining of GPNMB with ionized calcium-binding adaptor molecule 1, a marker of activated microglia, and with CD68, a Kupffer cell marker, will be needed. A third limitation is that the target molecule of HP-β-CD, when it showed preventive effects against NPC manifestations and an effect of reducing GPNMB expression, remains unclear. HP-β-CD is known as a solubilizing agent for lipophilic compounds and shows high affinity with cholesterol in biological systems [23]. Formation of the cholesterol-HP-β-CD complex has been considered central to the mechanism of correction of NPC manifestations. However, whether cholesterol is the true target molecule of HP-β-CD in the attenuation of NPC disease conditions has remained controversial, and Davidson et al. [5] suggested that HP-β-CD may interact with other NPC disease-related substrates, such as sphingolipids. Indeed, we previously demonstrated that HP-β-CD can significantly attenuate sphingomyelin accumulation in *Npc1*-deficient Chinese hamster ovarian cells, as well as the accumulation of free cholesterol [24]. Marques et al. [25] reported that glucocerebrosidase 2 gene knockout and pharmacological inhibition ameliorated the manifestations of NPC, such as Purkinje cell loss, motor dysfunction, and shortened lifespan, without correcting cholesterol levels, in *Npc1*^−/−^ mice. In addition, they demonstrated that miglustat inhibited GPNMB expression induced by U18666A, an NPC-phenotype-inducer, in RAW264.7 macrophages [13]. Further studies to clarify the target molecule of HP-β-CD in the amelioration of disease and reduction of GPNMB in NPC will be needed. As a fourth limitation, the mechanisms of hepatoprotection and reduction of GPNMB levels in liver and serum by intracerebroventricular HP-β-CD treatment remains unknown. The dose of HP-β-CD injected through the intracerebroventricular route, 30 mg/kg in this study, was less than one-hundredth the dose in a study of systemic subcutaneous treatment (4000 mg/kg, every week), so we postulated that HP-β-CD applied intracerebroventricularly might act locally in the brain. Indeed, we previously demonstrated that 400 mg/kg HP-β-CD subcutaneously administered every week had no effects on hepatic disorder, such as serum ALT elevation and histological changes, in *Npc1*^−/−^ mice [7]. We considered that a full dose of HP-β-CD injected intracerebroventricularly (30 mg/kg) would exert no effects on liver manifestations if it would be distributed to peripheral organs. Interestingly, Vite et al. [16] also reported that intracisternal HP-β-CD can significantly reduce serum ALT elevation in *Npc1*^−/−^ mice. Therefore, intracerebroventricular or intracisternal treatment with HP-β-CD should attenuate peripheral organ disorder in NPC. As the mechanisms behind this remain unknown, further basic and clinical studies are warranted. As a final limitation, whether GPNMB could be a predictive factor of prognosis in NPC remains unclear. In this study, we found that GPNMB expression in serum, cerebellum, and liver was decreased, accompanied by the attenuation of Purkinje cell loss and liver dysfunction by intracerebroventricular HP-β-CD treatment. In addition, although we demonstrated that repeated intracerebroventricular treatment with HP-β-CD drastically attenuated the shortened lifespan of *Npc1*^−/−^ mice, the relationship between changes in GPNMB and prognosis was not evaluated in this study. Further study will be needed to shed light on this important issue.

In conclusion, we demonstrated that serum GPNMB was responsive to the attenuation of NPC manifestations by intracerebroventricular treatment with HP-β-CD in an NPC model mouse. Our results suggest that serum GPNMB is a potential biomarker for assessing the disease condition during HP-β-CD treatment, although further basic and clinical studies of this are needed.

## 4. Materials and Methods

### 4.1. Reagents

HP-β-CD (average molecular weight: 1402.38, degree of substitution: 4.61) was kindly provided by Nihon Shokuhin Kako Co., Ltd. (Tokyo, Japan). Four percent buffered paraformaldehyde (PFA) phosphate buffer solution, which was used for sample storage, was purchased from Wako Pure Chemical Industries, Ltd. (Osaka, Japan). Deionized and distilled biopure-grade water was used throughout the study. All other reagents and solvents used were of reagent grade.

### 4.2. Animal Experiments

A total of 48 wild-type and *Npc1*^−/−^ age-matched mice were used: four wild-type and 14 *Npc1*^−/−^ mice were used to measure cerebellar GPNMB levels, while eight wild-type and 22 *Npc1*^−/−^ mice were used for the remaining analysis. The animal experiments were conducted at the Department of Clinical Chemistry and Informatics, Graduate School of Pharmaceutical Sciences, Kumamoto University. Male and female wild-type and *Npc1*^−/−^ mice were bred and kept at the Center for Animal Resources and Development, Kumamoto University. The *Npc1*^−/−^ mice were maintained under controlled conditions at constant room temperature (24 °C) with a 12-h light/dark cycle, and food and water were supplied ad libitum. The mice were weighed weekly until histological analysis was performed. The brain samples were collected at 8 weeks of age, on which immunohistochemical staining for calbindin was performed. All experiments complied with the animal use guidelines of the Committee for Ethics on Animal Experiments of Kumamoto University (approval numbers A27-133, A29-134, and A2019-103).

#### 4.2.1. Intracerebroventricular Administration of HP-β-CD in Npc1-Deficient Mice

The HP-β-CD solution for intracerebroventricular administration was adjusted as shown below. HP-β-CD was dissolved in water and vortexed so that the powder was completely solubilized, and the concentration was adjusted to 320.8 mM using a volumetric flask. The HP-β-CD solution was also adjusted to pH 7.2–7.4 and subjected to sterile filtration. After receiving intraperitoneal injection of the three types of mixed anesthetic agents (0.3 mg/kg medetomidine, 4.0 mg/kg midazolam, and 5.0 mg/kg butorphanol), the mice were fixed in a stereotaxic instrument for small animals (IMPACT-1000C and STEREOTAXIC INJECTOR KDS 310 Plus; Muromachi Kikai Co., Ltd., Tokyo, Japan). A needle was inserted through the left lateral ventricle into a burr hole in the skull and injected with saline or HP-β-CD. Heating was applied to maintain the body temperature of the mice at 37 °C during the surgery. The mice were given HP-β-CD twice intracerebroventricularly at 4 and 6 weeks of age. Weight changes were recorded once a week from 4 to 8 weeks of age, and samples were collected at 8 weeks and 3 days of age.

#### 4.2.2. Evaluation of Purkinje Cell Loss in the Cerebellum of Npc1-Deficient Mice

The effects of intracerebroventricular HP-β-CD on the neurodegeneration of NPC were assessed using Purkinje cells as an indicator of central nervous system dysfunction in the cerebellum. Mice were sacrificed after they had reached 8 weeks and 3 days of age. Under anesthesia, the brain was collected by perfusion with 4% PFA in phosphate-buffered saline. The brain samples were fixed in 4% buffered PFA at 4 °C and paraffin-embedded sections were prepared. Microtome sections prepared to 3 μm in thickness were incubated at 4 °C overnight with anti-calbindin D28K antibody (N-18) (Santa Cruz Biotechnology Inc., CA, USA; 1:100 dilution), which is a marker of Purkinje cells. The incubated sections were stained with Histofine^®^ SimpleStain MAX PO (Nichirei, Tokyo, Japan) and Mayer’s hematoxylin. Histopathological changes were imaged using a Biorevo BZ-9000 microscope system (Keyence Co., Osaka, Japan) and the number of calbindin-positive cells expressed throughout the cerebellum was measured.

#### 4.2.3. Quantification of GPNMB Levels of Npc1-Deficient Mice

GPNMB levels in mouse serum, liver, and cerebellum were assayed using the commercially available Mouse Osteoactivin/GPNMB ELISA (catalog number: DY2330), set in accordance with the manufacturer’s instructions (R&D Systems, Minneapolis, MN, USA). We also performed GPNMB immunohistochemical staining using Mouse Osteoactivin/GPNMB antibody (catalog number: BAF2330, R&D Systems) in liver and cerebellum samples and evaluated under microscopic condition using Biorevo BZ-9000 microscope system.

#### 4.2.4. Evaluation of Hepatic Changes in Npc1-Deficient Mice

To examine the effect of intracerebroventricular HP-β-CD on the hepatic dysfunction occurring in *Npc1*^−/−^ mice, serum transaminase levels and liver pathological changes were evaluated. Blood was collected from the inferior vena cava. The blood samples were centrifuged at 3000× *g* for 10 min at 4 °C and the serum was then collected. Serum ALT levels were measured using an automated analysis device (FUJI DRI-CHEM 7000 V; FUJIFILM Corporation, Tokyo, Japan). Liver samples were immediately weighed and some of the liver lobes were frozen in liquid nitrogen and stored at −80 °C for the measurement of GPNMB level. The other liver lobes were immediately fixed in 4% PFA, made into paraffin-embedded sections, and cut into 4-μm-thick slices for H&E staining. Histopathological images were obtained using Biorevo BZ-9000 microscope system.

#### 4.2.5. Evaluation of Lifespan in Npc1-Deficient Mice

We evaluated the effect of repeated treatment of HP-β-CD through the intracerebroventricular route on the lifespan in *Npc1*^−/−^ mice. We started the injection of HP-β-CD (30 mg/kg) at 4 weeks of age, in accordance with the method described in Section 4.2.1. The administration was then repeated every 2 weeks with the same dose and method, and general symptoms and body weight were observed to evaluate survival time. The survival times were recorded based on the day when the endpoint was reached, defined as death or an inability to consume food or drink independently.

### 4.3. Statistical Analysis

Statistical analyses were conducted using GraphPad Prism ver. 7.01 (GraphPad Software, San Diego, CA, USA). To examine the statistical significance of the results, multiple comparisons were performed. One-way ANOVA was used to test for the presence of a statistically significant difference, when uniform variance in the results was confirmed by Bartlett analysis (*p* < 0.05). If a significant difference (*p* < 0.05) was confirmed, the results were further analyzed for significant differences among groups using Tukey–Kramer’s multiple range test. If nonuniform variance was identified, nonparametric multiple comparison was conducted. Kruskal–Wallis analysis was used to confirm significant differences (*p* < 0.05), and then Dunn’s multiple test was applied as the post-hoc test. Survival data were analyzed using the Kaplan–Meier method, and the log-rank test was used to compare statistical significances.

## Data Availability

The datasets generated or analyzed during the current study are available from the corresponding author on reasonable request.

## Supplementary Materials

The following are available online at https://www.mdpi.com/1422-0067/22/1/452/s1. Figure S1. Quantification of glycoprotein nonmetastatic melanoma protein B (GPNMB) expression after intracerebroventricular 2-hydroxypropyl-β-cyclodextrin (HP-β-CD) treatment in *Npc1*^−/−^ mouse liver.

## Abbreviations

NPC	Niemann–Pick disease type C
HP-β-CD	2-Hydroxypropyl-β-cyclodextrin
WT	Wild type
ALT	Alanine aminotransferase
AST	Aspartate aminotransferase
H&E	Hematoxylin & eosin
IU/L	International unit per liter
