# Supplementary material for: Intracerebroventricular Treatment with 2-Hydroxypropyl-β-Cyclodextrin Decreased Cerebellar and Hepatic Glycoprotein Nonmetastatic Melanoma Protein B (GPNMB) Expression in Niemann–Pick Disease Type C Model Mice
Source: Int J Mol Sci. 2021 Jan 5;22(1):452. doi: 10.3390/ijms22010452 (PMC7795151; doi:10.3390/ijms22010452)
Supplement: Supplementary file 1 [file ijms-22-00452-s001.pdf]

**Supplementary Data**

FS1. Quantification of GPNMB positive cells in immunohistological images of Npc1$^{-/-}$ mouse liver.

GPNMB positive cells were quantified in seven to eleven random fields of each immunostained sample as shown in Supplemental Figure 1S.

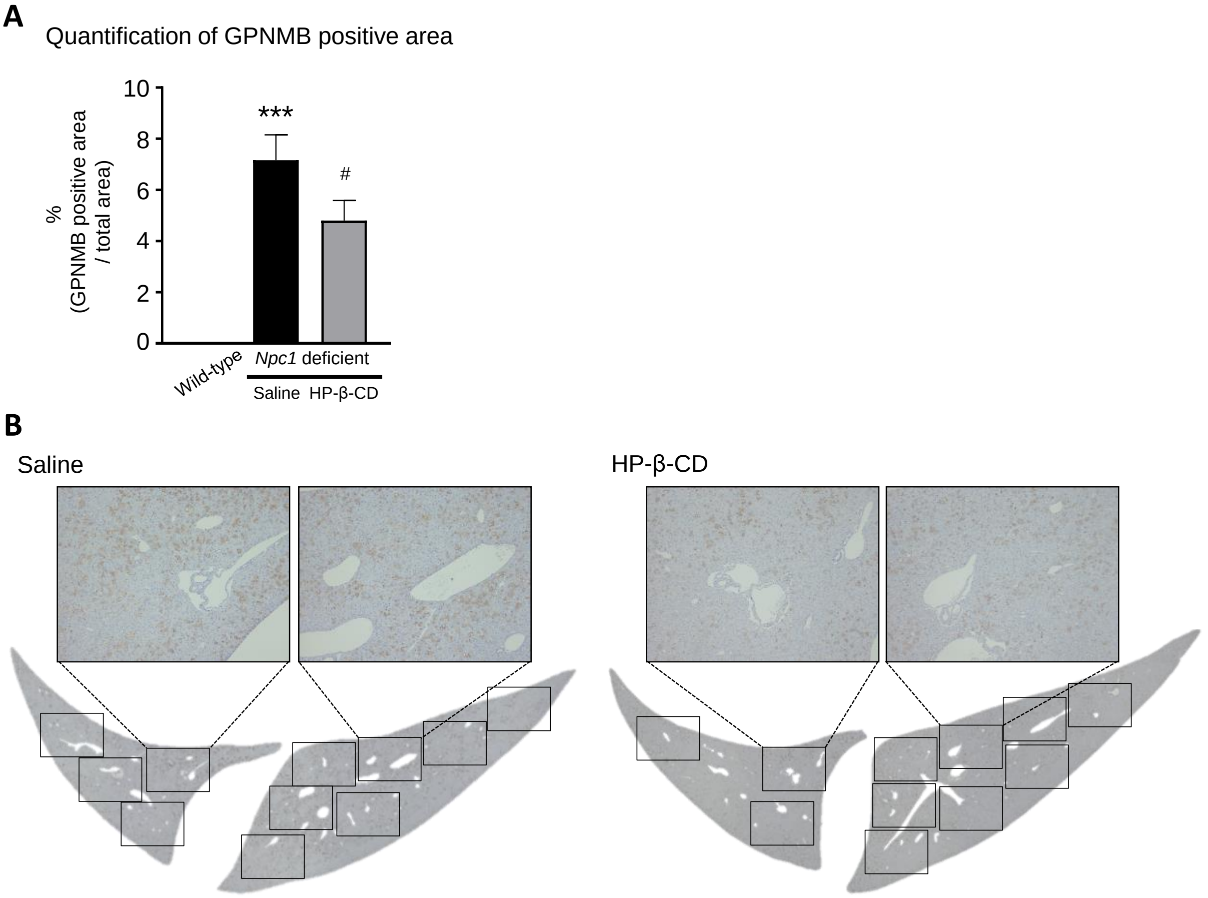


Figure S1. Quantification of glycoprotein nonmetastatic melanoma protein B (GPNMB) expression after intracerebroventricular 2-hydroxypropyl-β-cyclodextrin (HP-β-CD) treatment in *Npc1*$^{-/-}$ mouse liver. We took 7-14 random images from each liver sample and quantified the GPNMB expression. The quantification of the GPNMB positive cells was performed using Image J software. Each bar represents the mean ± S.E.M. ($n$ = 8-11). *** $p < 0.001$ compared with the wild-type group. # $p < 0.05$ compared with the saline group.
